# Sequestration of Late Antigens Within Viral Factories Impairs MVA Vector-Induced Protective Memory CTL Responses

**DOI:** 10.3389/fimmu.2019.02850

**Published:** 2019-12-04

**Authors:** Sha Tao, Ronny Tao, Dirk H. Busch, Marek Widera, Heiner Schaal, Ingo Drexler

**Affiliations:** ^1^Institute for Virology, Düsseldorf University Hospital, Heinrich-Heine-University, Düsseldorf, Germany; ^2^Institute of Microbiology, Immunology and Hygiene, Technical University Munich, Munich, Germany

**Keywords:** CTL, antigen presentation, Modified Vaccinia Virus Ankara (MVA), memory T cell activation, viral vector vaccine, viral immunology

## Abstract

Cytotoxic CD8+ T cell (CTL) responses play an essential role in antiviral immunity. Here, we focused on the activation of CTL which recognize epitopes derived from viral or recombinant antigens with either early or late expression kinetics after infection with Modified Vaccinia Virus Ankara (MVA). Late antigens but not early antigens failed to efficiently stimulate murine CTL lines *in vitro* and were unable to activate and expand protective memory T cell responses in mice *in vivo*. The reduced or absent presentation of late antigens was not due to impaired antigen presentation or delayed protein synthesis, but was caused by sequestration of late antigens within viral factories (VFs). Additionally, the trapping of late antigens in VFs conflicts with antigen processing and presentation as proteasomal activity was strongly reduced or absent in VFs, suggesting inefficient antigen degradation. This study gives for the first time a mechanistic explanation for the weak immunogenicity of late viral antigens for memory CTL activation. Since MVA is preferentially used as a boost vector in heterologous prime/boost vaccinations, this is an important information for future vaccine design.

## Introduction

Cytotoxic CD8+ T cell (CTL) responses play an essential role in antiviral immunity by eliminating infected cells after recognition of virus-derived peptide ligands on the cell surface (1). In contrast to virus-specific antibodies that can neutralize viruses by recognizing limited structural components on the surface of the pathogen, CD8+ T cell responses can essentially be directed against any viral protein that is produced within an infected cell as long as it contains epitopes presented by MHC class I. Thus, there is an increasing interest in developing vaccines that can raise efficient antiviral CD8+ T cell responses (2).

Modified Vaccinia Virus Ankara (MVA) is a highly attenuated strain of vaccinia virus (VACV) that has lost ~15% of the VACV genome, along with the ability to replicate in most mammalian cells (3). It has accumulated an impressive safety and immunogenicity profile in both preclinical and clinical studies, and is being actively explored as a promising vaccine vector for a number of infectious diseases and malignancies (4, 5). However, more knowledge is needed on how MVA as a replication deficient vector vaccine interacts with the host immune system, especially with dendritic cells (DCs), to induce strong immune responses (6–9).

The expression of target genes in poxvirus vectors is dependent on the use of native or synthetic poxvirus-specific promoters. Native gene expression kinetics of poxvirus has been initially described as early, intermediate and late (10) and further characterized by RNA deep sequencing (11). Some genes have elements of early and late promoters in their upstream region, giving rise to a fourth class, referred to as early/late. A fifth class of genes was revealed in 2008 exhibiting immediate-early expression (12). Next to strictly early (e.g., PK1L) or late prompters (e.g., P11), the expression of recombinant genes is most often driven by promoters with both, early and late activity (e.g., P7.5 or PH5) (13–16). The expression cassette is inserted either into one of the sites of the large genomic deletions in MVA or in the TK locus (17).

After infection, immediate-early and early gene transcription starts as soon as the core is released into the cytoplasm, while intermediate and late phases of gene expression occur in subcellular compartments, so-called viral factories (VFs), which are known to be the site of DNA replication followed by intermediate and late viral transcription and translation (18).

It has been shown that the level of gene expression from poxvirus vectors may correlate with the magnitude of the immune responses in MVA (14). Genes expressed early during infection tend to be preferentially associated with CD8+ T cell responses, while intermediate and/or late gene products appear to be favored targets for CD4+ T cell and antibody responses (19–23). This relative dichotomy is ill understood and may be correlated with the expression kinetics during infection (i.e., early vs. late) or to the nature of the antigens (i.e., transcription factor vs. structural protein). A previous report suggests that the sequestration of vaccinia viral late proteins in factories contributes to reduced cross-presentation (24). In this report, we used a set of MVA antigens expressed either by native or synthetic promoters with early or late activity. Since various vaccinia virus strains may display differences in their gene expression profiles, affecting sometimes the same gene, we confirmed the expression class for all relevant MVA genes by qPCR as has been shown for other vaccinia virus strains (12).

Using the attenuated VACV strain MVA, we demonstrated in the past that primary CTL responses against MVA-produced antigens were dominated by cross-priming *in vivo*, while infected professional antigen presenting cells (pAPC), such as DC, failed to induce primary CTL (8, 25). In the recall, we found that the immunodominance pattern was shaped by competing CTL (26). Thereby, the outcome of T cell competition in secondary responses strongly depended on the timing of viral antigen expression in infected APC, which was particularly characterized by poor proliferation of T cells recognizing epitopes derived from late viral proteins. Therefore, we anticipated that the timing of gene expression after MVA infection has a strong impact on viral T cell epitope presentation and processing (27).

In this study, we used a set of CTL lines in order to compare early and late viral antigen presentation kinetics and monitored the intracellular processing of MVA-delivered antigens in infected cells. Our results demonstrate that all late viral antigens investigated were initially localized in VFs. The trapping of late viral or recombinant antigens in VFs was associated with an impairment of proteasomal activity in this compartment, indicating protection of viral antigens from proteasomal degradation, which most likely accounts for the poor antigen processing and the delayed or even completely absent activation of CTL directed against late viral antigens. Furthermore, the reactivation of memory CD8+ T cells specific for recombinant antigen was almost abrogated, if the antigen was expressed late and resulted in complete loss of protection in a bacterial challenge model.

## Materials and Methods

### Mice and Vaccination

C57BL/6 mice were purchased from Janvier. HLA-A^*^0201 transgenic HHD mice (28) were derived from in-house breeding “Zentrale Einrichtung für Tierforschung und wissenschaftliche Tierschutzaufgaben (ZETT)” under specific pathogen-free conditions following institutional guidelines. Animal experiments have been conducted according to the German Animal Welfare Act (Tierschutzgesetz) and have been approved by the regional authorities (District Government of Upper Bavaria, Germany and the North Rhine-Westphalia State Environment Agency-LANUV NRW, Germany). Female mice between 8 and 12 weeks' old were used.

For MVA prime-boost studies, mice were vaccinated i.p. with 10^8^ IU recombinant or wildtype MVA, and sacrificed on the indicated days after vaccination. Splenocytes were analyzed by ICS/FACS as described below. For generation of CTL, C57BL/6 mice were vaccinated i.p. with 10^8^ IU MVA-Pe/l-OVA and sacrificed at day 8 post vaccination. Splenocytes were cultured for generation of CD8+ T cell lines as described below.

### Cell Lines

HeLa (CCL-2) and E.G7-OVA (CRL-2113) and UMNSAH/DF-1 (CRL-12203) cells were purchased from American Type Culture Collection (ATCC). All cells were cultured in RPMI 1640 medium (Invitrogen) containing 10% FBS (PAN Biotech) at 37°C in 5% CO_2_.

### Viruses

Modified Vaccinia Virus Ankara (MVA, cloned isolate F6) at 582nd passage on chicken embryo fibroblasts (CEF) and recombinant MVA (recMVA) were generated by homologous recombination as previously described (29). MVA was routinely propagated and titrated on DF-1 cells. Purification was performed by two successive ultracentrifuge sucrose cushions.

MVA-Pe-OVA or -Pl-OVA or -Pe/l-OVA viruses express the full length ovalbumin (OVA) gene under the respective early PK1L (Pe) or late promoter P11 (Pl) or early/late promoter P7.5 (Pe/l) (15, 26). MVA-Pl-OVA-mCherry encodes mCherry fused to OVA under the P11 late promoter. MVA-Pe-H3-eGFP or –Pl-H3-eGFP viruses express a mutant form of viral membrane protein H3 [with an inactivated early stop sequence at bp position 327 (TTTTTTT into TTTCTTT)] fused with an enhanced form of green fluorescent protein (eGFP) under control of the respective early or late promoter. MVA-Pe/l-Np-SIIN-eGFP viruses express a fusion gene encoding the nucleoprotein (Np) from influenza A virus (type Puerto Rico 68), the peptide SIINFEKL (OVA_257−264_) derived from OVA and eGFP expressed under control of the early/late P7.5 (Pe/l) promoter.

Recombinant MVA for A6 and B5 fusion proteins were generated using a BAC en passant recombination technique (30). MVA-Pe-A6-SIIN-mKate or –Pl-A6-SIIN-mKate express the viral gene A6 fused to the OVA_257−264_ peptide SIINFEKL followed by the fluorescent marker mKate2 under control of either the K1L early promoter (MVA-Pe-A6-SIIN-mKate, A6 with an inactivated viral early stop sequence) or the natural A6 late promoter (MVA-Pl-A6-SIIN-mKate), respectively. MVA-B5-OVA and MVA-B5-ZsYellow express the B5 gene fused to either OVA or ZsYellow1 under control of the natural B5 late promoter. VACV-Pe/l-Np-SIIN-mCherry [kindly provided by JW Yewdell (NIH)] producing influenza nucleoprotein fused to the SIINFEKL epitope followed by mCherry under control of the P7.5 early/late promoter. All viruses are listed in Table 1.

**Table 1:** recMVA constructs.

**Virus**	**Promoter**	**Target gene**	**Target epitope**	**Insertion site**
MVA-Pe-OVA	PK1L (early)	OVA	SIINFEKL (OVA_257−264_)	del III
MVA-Pl-OVA	P11 (late)	OVA	SIINFEKL (OVA_257−264_)	del III
MVA-Pe/l-OVA	P7.5 (early and late)	OVA	SIINFEKL (OVA_257−264_)	del III
MVA-Pl-OVA-mCherry	P11 (late)	OVA-mCherry	/	del III
MVA-Pe-H3-eGFP	PK1L (early)	H3-eGFP	/	del III
MVA-Pl-H3-eGFP	P11 (late)	H3-eGFP	/	del III
MVA-Pe-A6-SIIN-mKate	PK1L (early)	A6-SIIN-mKate	SIINFEKL (OVA_257−264_)	del VI
MVA-Pl-A6-SIIN-mKate	Natural A6 (late)	A6-SIIN-mKate	SIINFEKL (OVA_257−264_)	Natural A6 locus
MVA-B5-OVA	Natural B5 (late)	B5-OVA	SIINFEKL (OVA_257−264_)	Natural B5 locus
MVA-B5-ZsYellow	Natural B5 (late)	B5-ZsYellow	/	Natural B5 locus
MVA-Pe/l-GFP	P7.5 (early and late)	GFP	/	del III
MVA-Pe/l-Np-SIIN-eGFP	P7.5 (early and late)	Np-SIIN-eGFP	/	del III
VACV-Pe/l-Np-SIIN-mCherry	P7.5 (early and late)	Np-SIIN-mCherry	/	TK locus

### Generation of BMDC

Bone marrow derived dendritic cells (BMDC) were isolated by flushing femurs with cell culture medium. Erythrocytes were lysed in TAC buffer (0.144 M NH_4_Cl and 0.017 M Tris pH 7.65) (Sigma-Aldrich) in a 37°C water bath for 2 min under shaking conditions. Thereafter, cells were washed and filtered through a 100 μm cell strainer. Within 94 × 16 mm petri dishes, 5 × 10^6^ cells were seeded in and cultivated with 10 ml RPMI1640 containing 10% FCS, 50 μM 2-ME (2-mercaptoethanol) and 10% GM-CSF (conditioned medium obtained as supernatant from B16 cells expressing GM-CSF; a kind gift from Georg Häcker, University of Freiburg, Germany). Cultures were incubated at 37°C. On day 3, additional fresh 10 ml medium containing 10% GM-CSF was added. On day 6, 10 ml medium was replaced by fresh medium containing 10% GM-CSF. Cells were collected and used on day 7.

### Generation and Maintenance of Antigen-Specific CTL

All peptides were purchased from Biosynthan: A19L_47_ (K^b^), B8R_20_ (K^b^), OVA_257_ (K^b^), A6L_6_ (A2), B22R_79_ (A2), I1L_211_ (A2), H3L_184_ (A2). Peptides were diluted in DMSO (1 mg/ml). For loading of APC, peptides were sonicated for 30 s and used at a concentration of 1 μg/ml.

For generating CTL, LPS-blasts were prepared by incubating 1 × 10^6^ cells/ml splenocytes from naïve C57BL/6 mice with 25 μg/ml LPS (Sigma-Aldrich) and 7 μg/ml dextran-SO_4_ (Sigma-Aldrich). Cells were cultured in a T75 flask with 40 ml at 37°C, 5% CO_2_ and 90% humidity. On day 4, cells were harvested, irradiated with 30 Gy and washed with RPMI 1640 medium. For each peptide, LPS-blasts were resuspended in 1 ml RPMI 1640 medium containing 5 μg/ml ß-2 microglobulin and 1 ng/ml peptide. Cells were incubated for 30 min and washed with cell culture medium. In each well of a 24-well-plate, 3 × 10^6^ LPS-blasts and 7 × 10^6^ splenocytes from MVA-Pe/l-OVA vaccinated C57BL/6 mice were co-cultivated for 8 days.

For maintenance, T cells were restimulated weekly using peptide loaded EL.4 cells irradiated with 100 Gy and naïve splenocytes in medium containing 5% T cell growth factor (TCGF) [conditioned medium as supernatant from rat splenocytes stimulated with 5 μg/ml Concanavalin A, as previously described (31)]. For restimulation of HLA-A2.1-restricted CTL generated from HHD mice, JA2.1 cells (a kind gift from Matthias Theobald, University of Mainz, Germany) were used instead of EL.4 cells.

### Antigen Presentation Assays

BMDC were infected for indicated hours at MOI 10 and washed with cell culture medium. Thereafter, 4 × 10^5^ infected BMDC were co-cultured with 2 × 10^5^ antigen-specific CTL per well in the presence of 1 μg/ml BFA (Sigma-Aldrich) for 4 h in 96-well-plate at 37°C in a humidified atmosphere containing 5% CO_2_. Uninfected BMDC or those pulsed with 1 ng of irrelevant peptide were used as negative controls in order to assess background activity of T cells. BMDC pulsed with the respective cognate peptide for the CTL lines were used as positive controls.

OVA-expressing E.G7-OVA cells were incubated with ice-cold acid stripping buffer (131 mM sodium citrate, 66 mM sodium phosphate, and 1% BSA pH 3) for 2 min in order to remove surface MHC molecules, then washed with medium and infected with MVA (MOI 10) or mock for indicated hours, modified from Loi et al. (32). After the incubation time, cells were washed and surface staining for *de novo* synthesized MHC class I was performed at 4°C with anti-H-2K^b^ or anti-SIINFEKL/K^b^ antibodies and Aqua viability dye.

### Spleen and Blood Preparation and CD8+ T Cell Analysis

For *ex vivo* CD8+ T cell analysis, **s**pleens were removed from MVA-vaccinated mice and homogenized with a syringe plunger over metal grid with cell culture medium. Erythrocytes were lysed with 3 ml TAC buffer and washed. Cells were filtered by 70 μm cell strainer and counted. For T cell restimulation, 4 × 10^6^ splenocytes were further incubated with respective peptides (1 μg/ml) for 5 h in the presence of BFA. IFNg was stained with specific antibodies (ICS).

For tetramer staining, blood was taken from MVA-OVA vaccinated mice. Erythrocytes were lysed with TAC buffer. After washing, PBMC were further discriminated for viability with dye (Invitrogen) and stained with PE-conjugated H-2K^b^/OVA_257_ tetramers (kindly provided by D. H. Busch, Institute of Microbiology, Technical University Munich).

### ICS and Flow Cytometry

ICS (intracellular cytokine staining) was performed as described earlier (15). Briefly, cells were washed with FACS buffer and then stained with 1 μg/ml ethidium monoazide bromide (Life Technologies GmbH) on ice under bright light for 20 min to mark dead cells. Surface markers stained by anti-CD8α antibodies (APC anti-CD8α, 5H10, Invitrogen) for 30 min. Cells were then fixed and permeabilized according to the manufacturer's protocol (BD Cytofix/Cytoperm™ Kit). Cells were stained with anti-IFNg antibodies (FITC anti-IFNg, XMG1.2, BD) for 30 min. Finally, cells were fixed with 1% PFA and used for FACS analysis.

For detection of SIINFEKL/K^b^ complexes at the cell surface, anti-SIINFEKL/K^b^ APC antibody (eBioscience 25-D1.16) was used after CD16/32-Fc-blockade (2.4G2, BD) and viability dye (Invitrogen).

FACS analysis was performed on BD FACS CantoII and FlowJo 6.4.2 software.

### Confocal Microcopy

Adherent cells were grown and infected in microscope dishes or chambers. Cells were washed and fixed with 4% PFA for 15 min. If intracellular staining was needed, cells were permeabilized with 0.25 % Triton X-100 for 3 min. In order to block unspecific binding, cells were incubated in PBS containing 5% BSA or FCS for 1 h at room temperature. Primary and secondary antibodies are performed at room temperature for 1 h. At last, cells were washed and kept in PBS at 4°C. Before analysis, DAPI (Invitrogen) was added to the cells for 10 min.

Following antibodies were used: rabbit anti-calnexin antibody (sigma C4731) and anti-rabbit Alexa Fluor 594 IgG (H+L) (Invitrogen) for ER staining; mouse anti-Golgi (GM130, sigma) and anti-mouse Alexa Fluor 647 IgG (H+L) (Invitrogen) for Golgi staining; 20S alpha 1+2+3+5+6+7 (abcam ab22674) for total proteasomes.The active proteasomes in infected cells were stained by using Proteasome Activity Probe (Me4BodipyFL-Ahx3Leu3VS, 500 nM, BostonBiochem), which is a cell permeable fluorescent substance that allows for accurate profiling of proteasomal activity in cell with high sensitivity (33).

HeLa cells have been transiently transfected with Proteasome Sensor Vector (pZsProSensor-1) plasmid encoding the gene for Zoanthus sp. Reef coral Green Fluorescent Protein (ZsGreen) fused to the mouse ornithine decarboxylase (MODC) degradation domain (amino acids 410–461) (Clontech #632425). This vector is designed for studies of proteasome function in mammalian cells. Since the MODC degradation domain targets the constitutively expressed protein for rapid degradation, the protein does not accumulate in cells until the proteasome is inhibited, which is indicated by an increase in green fluorescence.

Imagines were performed at the university image facility CAi (Center for Advanced Imaging, HHU, Düsseldorf) using confocal microscopy (Zeiss LSM 780 or LSM 710). Images were processed and analyzed with Fiji software.

### Bacterial Challenge and Determination of Bacterial Load

Recombinant *Listeria monocytogenes* expressing OVA (Lm-OVA) was kindly provided by Hans-Willi Mittruecker (UKE, Hamburg, Germany). In brief, vaccinated mice were challenged i.v. with 2 × 10^6^ CFU Lm-OVA. 3 days after the bacterial challenge, spleens were homogenized through 70 μm cell strainers and resuspended in 5 ml sterile PBS. Cell suspensions were diluted 1:10, 1:100, 1:1000 in 0.1% Triton X-100/PBS to release intracellular Lm-OVA from infected cells. Aliquots of 10 μl per dilution were plated in triplicates on BHI plates and incubated overnight at 37°C. Colony-forming units (CFU) of Lm-OVA were counted on the following day and calculated per organ according to the respective dilutions.

### Western Blot

For detection of viral derived proteins, BMDC were left uninfected or infected with indicated MVA (MOI 10) for indicated hours. Cell lysates, SDS-PAGE, nitrocellulose membranes were prepared as described earlier (15). Membranes were blocked with 5% BSA in Tris-buffered saline supplemented with 0.1% Tween-20 (TBST) for 1 h. Rabbit anti-OVA (abcam), rabbit anti-mKate (evrogen), mouse anti-GFP antibodies or mouse anti-ßactin (Sigma)were diluted in TBST and incubated with membranes for 1 h. Peroxidase-conjugated goat anti-rabbit or anti-mouse IgG (Jackson) was incubated with membranes for 1 h. Chemiluminescence detection was done with Super Signal West Dura Chemiluminescent Substrate (Thermo Scientific).

### Quantitative RT-PCR

The mRNA expression kinetics for MVA genes in infected BMDC (MOI 10) were determined between 0 and 6 h post infection (p.i.). Total RNA was isolated by RNeasy miniKit (Qiagen) according to the manufacturer's protocol and subsequently, 2 μg of total RNA were subjected to DNase digestion (RNase-free DNase Set) followed by cDNA synthesis (RevertAid H Minus First Strand cDNA Synthesis Kit, Thermo Scientific). The cDNA was amplified in duplicate with primers (eurofins Genomics) listed in Table 2 and SYBR™ Select Master Mix (Applied Biosystems). Amplification (1 cycle: 50°C for 2 min, 95°C for 10 min; 40 cycles: 95°C for 15 s, 60°C for 1 min) was monitored using Applied Biosystems 7500 Fast Real-Time PCR System. Relative expression was analyzed using 2–ΔCT method, where ΔCT = (Ct gene of interest—Ct internal control 18 s). Data are represented as fold change of mRNA in each time point relative to 0 h and are displayed as the means and SEM. All qRT-PCR experiments were performed in duplicates and repeated in at least three independent experiments.

**Table 2:** Primers used for qRT-PCR.

**Primer**	**Sequence**
18s rRNA fwd	AAA CGG CTA CCA CAT CCA AG
18s rRNA rev	CCT CCA ATG GAT CCT CGT TA
MVA-B8R fwd	ATC CGC ATT TCC AAA GAA TG
MVA-B8R rev	ACA TGT CAC CGC GTT TGT AA
MVA-B5R fwd	TGT CCT AAT GCG GAA TGT CA
MVA-B5R rev	AAC GCC ACC GAT AGA AAA TG
MVA-H3L fwd	GTC TTG AAG GCA ATG CAT GA
MVA-H3L rev	TCC CGA TGA TAG ACC TCC AG
MVA-A19L fwd	GCATGACGTGTTCTGCCT
MVA-A19L rev	GGCCAGTGTATTACCCCTCA
MVA-B22R fwd	CTCATTCCTCATCCACAACCCA
MVA-B22R rev	GACAGGGTCTGAACTGGGCA
MVA-A6L fwd	ACATCAATGCACAACAATTCGC
MVA-A6L rev	TCAATAGACATCCAACATCGACA
MVA-I1L fwd	CAGTGCCCGTGCATTGAAAG
MVA-I1L rev	TCTTGTGACCAACTCATCTACCA
MVA-D13L fwd	TCGTATCCAGGGTACTCACAAGA
MVA-D13L rev	TGGAACCTCAACAATCTCGGCA
OVA fwd	CACAAGCAATGCCTTTCAGA
OVA rev	GACTTCATCAGGCAACAGCA
mKate2 fwd	CCTTCGACATCCTGGCTACC
mKate2 rev	CTCTCCCATGTGAAGCCCTC
eGFP fwd	ACGTAAACGGCCACAAGTTC
eGFP rev	AAGTCGTGCTGCTTCATGTG
ZsYellow1 fwd	TGCGAGAAGATCATGCCCGT
ZsYellow1 rev	CCGTCCTTCAGCAGCAGGTA

### Statistical Analysis

All results are expressed as means and SEM (ICS *n* ≥ 3, IF *n* ≥ 30), either pooled or representative from at least three independent experiments. Image pictures are representative from three independent experiments. The statistical significance was tested by unpaired *t*-test (two-tailed) using GraphPad Prism 6. *P* < 0.05 were considered significant with further subdivision: ^*^P < 0.05, ^**^P < 0.01, ^***^P < 0.001, ^****^P < 0.0001.

## Results

### Presentation of Late Viral Antigens to CTL Is Strongly Impaired

In the VACV replication cycle, distinct subsequent phases of viral gene expression such as early (0–1.5 h p.i.) which includes the subclass immediate early (0–0.5 h p.i.), intermediate (1–3 h p.i.) and late (>3 h p.i.) may be distinguished (10, 22, 23). However, there may be strain-specific variations for some genes concerning the expression pattern due to genetic differences e.g., by mutation of genes in attenuated strains, like in MVA (3). In order to study the processing and presentation of MVA-derived antigens which are expressed at different time points during the infectious cycle, a set of early or late genes were selected encoding antigens which contain published epitopes allowing for studies on CD8+ T cell responses (Table 3). The expression profile of each gene investigated in this study was confirmed by qRT-PCR (Supplementary Figure 1).

**Table 3:** HLA-A2.1 (HHD)- and H-2K^b^/D^b^-restricted CTL specificities.

**MHC-restriction** **(mouse strain)**	**Antigen**	**Expression** **time**	**Specificity** **(Epitope)**
HLA-A2.1 (HHD)	B22	Early	B22R_79_
	I1	Late	I1L_211_
	H3	Late	H3L_184_
	A6	Late	A6L_6_
H-2K^b^/-D^b^ (C57BL/6)	B8	Early	B8R_20_ K^b^
	A19	Late	A19L_47_ K^b^
	D13	Late	D13L_118_ D^b^
	OVA	Early or late	OVA_257_ K^b^

A set of HLA-A2.1-restricted antigen-specific CTL lines which recognize the epitopes B22R_79_, H3L_184_, I1L_211_, and A6L_6_ derived from the respective MVA proteins B22 (early), H3 (late), I1 (late), and A6 (late) (Supplementary Figure 1 and Table 3),was generated after vaccination of HLA-A2.1-transgenic HHD-mice as described before (26).

**Figure 1:**
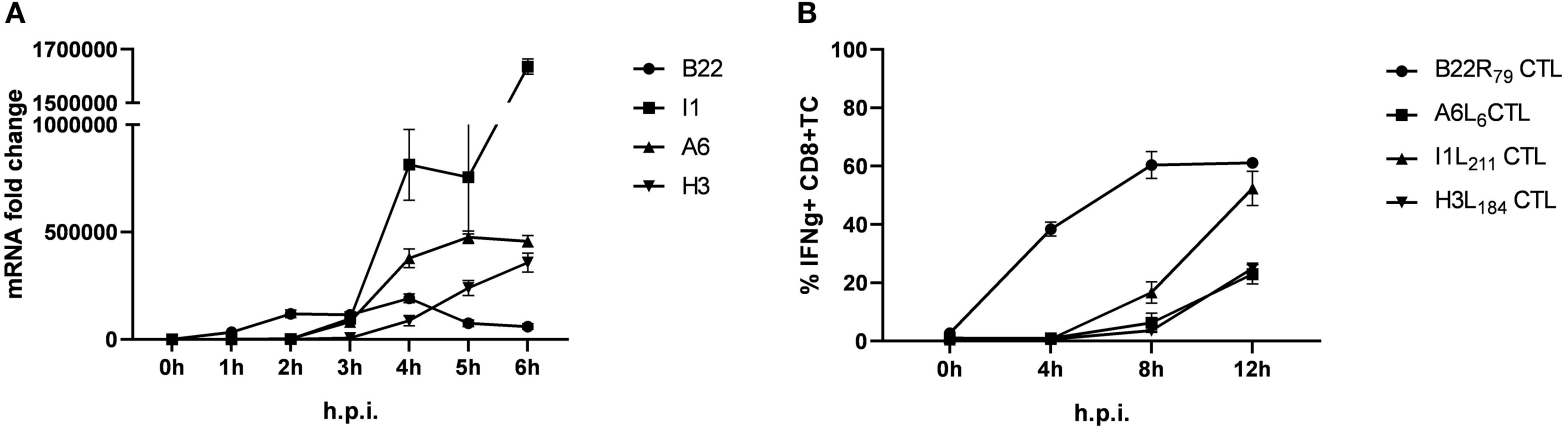
Presentation of viral late antigens to CTL is impaired. **(A)** qRT-PCR. Gene expression profile for B22, I1, A6, and H3 in BMDC infected with MVA-wt (MOI 10) from 0 to 6 h post infection (h p.i.). **(B)** Kinetic analysis of IFNg production [4 h intracellular cytokine staining (ICS)] by HLA-A2.1-restricted HHD-derived CD8+ T cell lines with the indicated epitope-specificities after stimulation with MVA-infected HLA-A2.1+ BMDC (MOI = 10) derived from HHD mice. The presentation of all late antigens tested (I1, A6, and H3) allowed for the activation of the respective CTL lines with a strong delay compared to CTL specific for early antigen B22. All data are means and SEM (*n* ≥ 3) from three independent experiments.

Using infected human lymphoblastoid B cell lines (LCL) as stimulators we have previously shown that some late antigens failed to activate CTL even 8 h post infection (p.i.) (26). To extend these findings, we used murine professional APC (HLA-A2.1+ BMDC from transgenic HHD mice) to monitor antigen presentation during MVA infection for an extended period of time (up to 12 h p.i.). In this setting, congenic HHD mouse derived HLA-A2.1-restricted CTL specific for the virally encoded early antigen B22 and the late antigens A6, I1 or H3 were used. As with human APC, despite the presence of late gene transcripts as early as 3 h p.i. (Figure 1A), the activation of all late viral antigen-specific CTL was strongly delayed when using BMDC (measurable around 8 h p.i.) (Figure 1B). Thus, we confirmed the impaired late antigen presentation to HLA-A2.1-restricted CTL with an additional APC subset.

To demonstrate that the above finding is not mouse strain-specific, a set of antigen-specific CTL lines was generated from C57BL/6 mice vaccinated with recombinant MVA (recMVA) viruses. These CTL recognize the viral late A19L_47_ and D13L_118_ as well as the early B8R_20_ epitope derived from respective viral proteins (A19, D13, B8) or the SIINFEKL epitope OVA_257_ derived from chicken ovalbumin (OVA). The expression kinetics of each gene was confirmed for MVA by qPCR (Supplementary Figures 1, 2A). To confirm CTL specificity and to exclude low affinity for respective epitopes, we performed peptide titration experiments which showed that all CTL were specifically activated following co-cultivation with respective peptide-pulsed APC as determined by IFNg production (Supplementary Figure 2B). Of note, A19L_47_-specific CTL showed highest avidity. Next, we used BMDC infected with MVA-wt to monitor the endogenous recognition of infected cells which was remarkably low for D13L_118_- and A19L_47_-specific CTL as compared to B8R_20_-specificCTL (Supplementary Figure 2C). Importantly, the unresponsiveness of A19L_47_-and D13L_118_-specific CTL was not due to a functional defect of T cells (Supplementary Figure 2B).

Taken together, HLA-A2.1- as well as H-2K^b^/D^b^-restricted CTL specific for epitopes derived from late-expressed viral antigens showed a strongly delayed and reduced activation profile by infected cells as compared to CTL specific for early antigens despite the presence and abundance of viral late gene transcripts.

### Unresponsiveness of Memory CD8+ T Cells Against Late Antigens *in vivo* Results in Loss of Protection Against Bacterial Challenge

Next, we wanted to investigate if the above findings have relevance for CD8+T cell responses *in vivo*. We used OVA as a model antigen expressed by recMVA under control of either an early (PK1L) or late (P11) promoter (MVA-Pe-OVA or -Pl-OVA). We first characterized these constructs *in vitro* and then used them as vaccines in immunization experiments to determine the frequency, functionality and protective capacity of the vaccine-induced T cell response.

**Figure 2:**
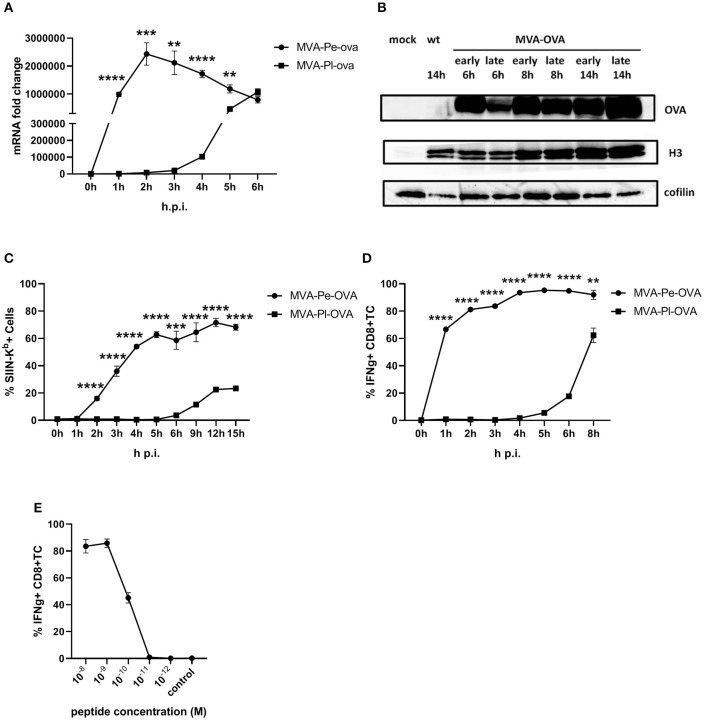
Presentation of OVA to OVA_257_-CTL is significantly delayed and reduced when expressed as a late antigen. **(A)** The mRNA levels for OVA were determined by qRT-PCR in C57BL/6 BMDC infected with MVA-Pe-OVA or MVA-Pl-OVA with MOI 10 between 0 and 6 h p.i. **(B)** Western blot analysis for OVA protein synthesis in cell lysates from BMDC infected with MVA-Pe-OVA or MVA-Pl-OVA harvested at the indicated time pints p.i. Viral protein H3 served as an infection control, cellular cofilin as a loading control. Anti-OVA, anti-H3, and anti-cofilin antibodies were used. **(C)** Kinetic analysis of SIINFEKL/K^b^ complex expression at the surface of MVA-Pe-OVA or MVA-Pl-OVA infected BMDC. Mouse anti-SIINFEKL/K^b^ APC antibody was used after the viability dye staining. Frequency of SIINFEKL/K^b^+ cells (% SIIN-K^b^+ cells) is shown. **(D)** Activation of OVA_257_-CTL from C57BL/6 BMDC infected with MVA-Pe-OVA or MVA-Pl-OVA for the indicated hours. ICS (4 h) for IFNg production in OVA_257_-specific CD8+ T cells is shown. **(E)** Peptide titration showed valid avidity for OVA_257_-CTL. BMDC were loaded with indicated peptides at concentrations ranging from 10^−8^ to 10^−12^ M or with irrelevant MHC class I binding peptide (control) at 10^−8^ M as negative control. ICS for IFNg production. All data are means and SEM (*n* ≥ 3) from three independent experiments. *P < 0.05; **P < 0.01; ***P < 0.001; ****P < 0.0001 (two-tailed Student's *t*-test).

The expression pattern of early or late OVA was confirmed on mRNA level by qPCR (Figure 2A) and protein level by WB (Figure 2B). In line with above findings, H-2K^b^+ BMDC from C57BL/6 mice infected with recMVA expressing OVA early (MVA-Pe-OVA) could present SIINFEKL/K^b^ complexes on their surface as early as 2 h p.i (Figure 2C). In contrast, late expressed OVA (MVA-Pl-OVA) resulted in strongly delayed surface expression of SIINFEKL/K^b^ complexes, first detectable around 6 h p.i (Figure 2C). MFI for SIINFEKL/K^b^ showed similar results (Supplementary Figure 3A). When measuring IFNg production of CTL lines *in vitro*, OVA-specific CTL were highly activated at 1 h p.i. (>60%) using BMDC infected with MVA-Pe-OVA, while these CTL were not stimulated at all before 4 h p.i. and did not even reach maximal cytokine production at 8 h p.i. using MVA-Pl-OVA (Figure 2D). Notably, almost comparable mRNA levels for OVA were detectable at 1 h p.i. for early OVA and 5 h p.i. for late OVA (Figure 2A). Peptide titration experiments indicated that OVA_257_-CTL were functional and specific (Figure 2E).

**Figure 3:**
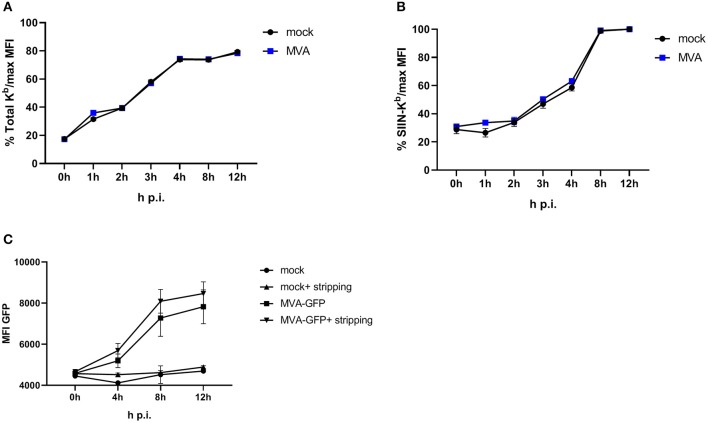
Antigen presentation machinery is not impaired in MVA-infected cells. OVA-expressing E.G7-OVA cells were incubated with ice-cold acid stripping buffer (131 mM sodium citrate, 66 mM sodium phosphate, and 1% BSA pH 3) for 2 min to allow for removal of surface MHC molecules from the cell surface. After washing with medium, stripped cells were infected with MVA-GFP under the control of the early/late promoter P7.5 (MVA-Pe/l-GFP) at MOI 10 or mock for the indicated hours. After respective incubation time, cells were washed and surface staining was performed with anti-H-2K^b^ Ab **(A)** or anti-SIINFEKL/K^b^ **(B)** after viability dye staining determining the *de novo* synthesis of peptide-loaded MHC class I molecules. **(C)** MFI of GFP indicated MVA-infected cells. All data are means and SEM (*n* ≥ 3) from three independent experiments.

To achieve efficient T cell activation, the MHC class I antigen presentation machinery of an APC must be fully functional. In order to exclude an impairment of the antigen presentation machinery due to the viral infection itself, we used an experimental setup which allows the exact quantification of newly surface expressed MHC I molecules as well as *de novo* peptide loading after infection with MVA [adapted from (32)]. E.G7-OVA cells were incubated with ice-cold acid stripping buffer (131 mM sodium citrate, 66 mM sodium phosphate, and 1% BSA pH 3) to wash away the remaining MHC molecules at the cell surface. As a consequence, only the newly synthesized MHC class I will be stained after MVA infection. We clearly show that the overall *de novo* H-2K^b^ surface expression (Figure 3A) as well as the amount of newly synthesized peptide/MHC I complexes (SIINFEKL/H-2K^b^) on the cell surface (Figure 3B) were comparable between mock- and MVA-GFP infected E.G7-OVA cells which stably express OVA. Determination of MFI for GFP served as an infection control (Figure 3C).

**Figure 4:**
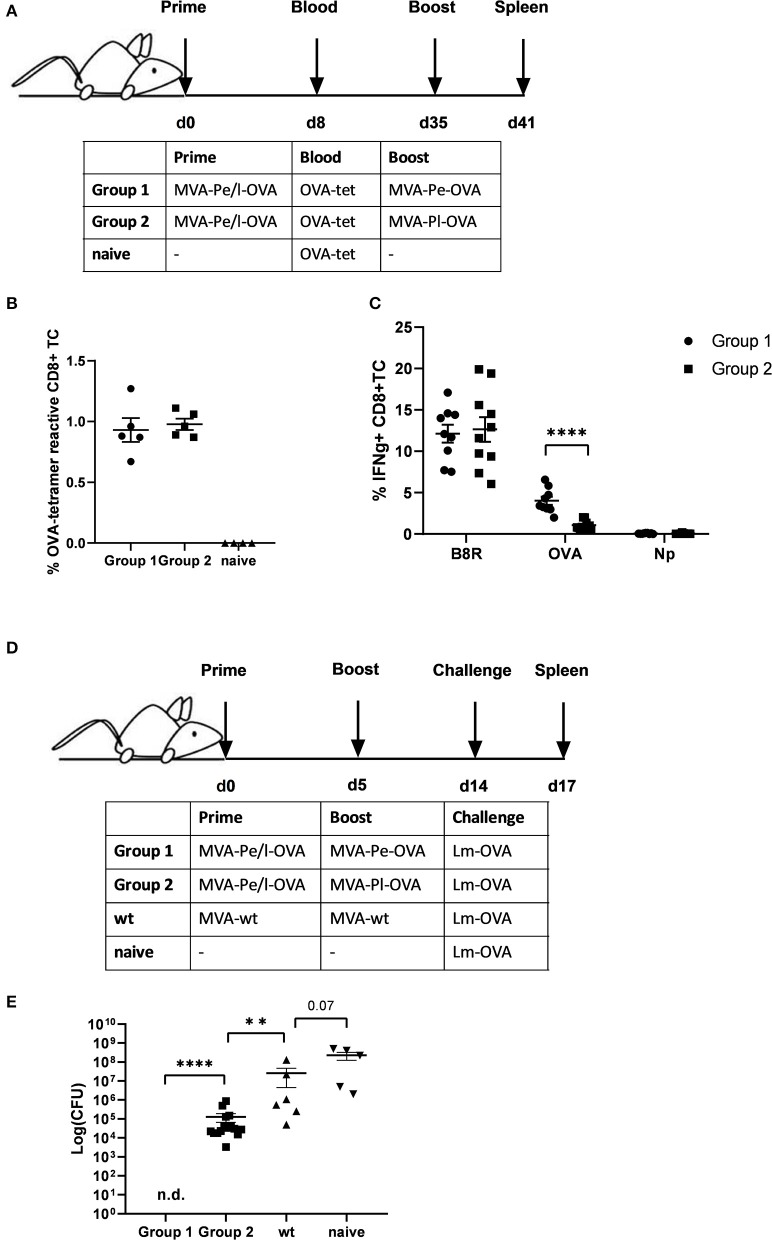
Reduced CD8+ T cell responses and loss of protection against a pathogen challenge *in vivo* when immunogenic antigens were expressed late by MVA vectors. **(A)** Scheme for heterologous MVA-prime-boost vaccination experiment in C57BL/6 mice. For groups 1 and 2, mice were primed i.p. with 10^8^ IU MVA-Pe/l-OVA expressing OVA early and late. On day 8, blood was taken for OVA-specific tetramer staining (results shown in **B**). On day 35, mice in group 1 were boosted with 10^8^ IU MVA-Pe-OVA (expressing OVA early) and in group 2 boosted with 10^8^ IU MVA-Pl-OVA (expressing OVA late). On day 41, spleens were taken and CD8+ T cell responses analyzed (results shown in **C**). **(B)** Priming. OVA_275_-specific tetramer staining of PBMC from mice primed with MVA-Pe/l-OVA (group 1 and 2) at 8 d p.i. or from uninfected mice (naive), demonstrated comparable OVA_275_-specific T cell frequencies in group 1 and 2 before boosting. **(C)** Boosting. On day 35 (memory phase), group 1 was boosted with MVA-Pe-OVA; group 2 with MVA-Pl-OVA. Six days later, ICS and FACS analysis was performed determining B8R- and OVA-specific IFNg production in splenocytes from both groups. Influenza nucleoprotein peptide (Np) served as negative control. **(D)** Scheme for bacterial challenge after heterologous MVA-prime-boost *in vivo*. Mice were primed on day 0 and boosted on day 5 with 10^8^ IU recMVA (group 1 and 2) or MVA-wt, or were left unvaccinated (naïve). Nine days later (day 14) all mice were challenged i.v. with 2 × 10^6^ CFU Lm-OVA. **(E)** Three days after the challenge (d17), the residual bacterial loads were determined as colony forming units (CFU) in the spleen. n.d. indicates not detectable. All data are means and SEM from three independent experiments. Each dot represents one mouse. *P < 0.05; **P < 0.01; ***P < 0.001; ****P < 0.0001 (two-tailed Student's *t*-test).

Given the evidence that late expression of OVA failed to timely and efficiently restimulate OVA-specific CTL *in vitro*, we wanted to test the ability of the two constructs to reactivate OVA-specific memory CTL *in vivo*. First, we vaccinated mice i.p. with MVA-Pe/l-OVA which expresses OVA early and late to allow for comparable memory T cell formation in all vaccines and subsequently boosted these mice at day 35 after priming (memory phase) with either MVA-Pe-OVA or MVA-Pl-OVA expressing OVA either early or late, respectively (Immunization scheme, Figure 4A). OVA-specific tetramer staining of PBMC revealed comparable amounts of OVA-specific CD8+ T cells in all mice after priming, which were divided into 2 groups for the following boost immunization (Figure 4B). At day 35 post prime, OVA was delivered in the boost vaccination by MVA vectors expressing the antigen either early (MVA-Pe-OVA; group 1) or late (MVA-Pl-OVA; group 2). As anticipated, expansion of OVA-specific CD8+ T cells was exclusively seen in MVA-Pe-OVA boosted mice, whereas OVA-specific CTL in MVA-Pl-OVA re-vaccinated mice were not activated and failed to proliferate (Figure 4C). Importantly, the T cell responses against the virus backbone antigen B8 were comparable for both MVA vaccines in the recall, suggesting efficient vaccination with either MVA vector. These data clearly indicate that the activation and expansion of memory CTL *in vivo* strongly depended on the timing of viral antigen expression resulting in a complete neglect of CTL specific for late antigens, most likely due to the impaired processing and presentation of the OVA epitope by infected APC.

Furthermore, the impaired CTL response influenced the protective capacity of MVA vaccines against a lethal challenge with a pathogen whose clearance depends mainly on CD8+ T cells (34). To this end, we were using a high dose of *Listeria monocytogenes* expressing OVA (Lm-OVA) to infect mice after prime-boost with MVA (Figure 4D). In this setting, only mice boosted with MVA-Pe-OVA were fully protected from the bacterial challenge. Mice boosted with MVA-Pl-OVA showed a reduced bacterial load compared to MVA-wt boosted mice, but allowed extensive Lm-OVA replication (Figure 4E).

### Distinct Localization of Early and Late Viral Antigens May Be Correlated to Efficacy of MHC Class I Presentation

**Figure 5:**
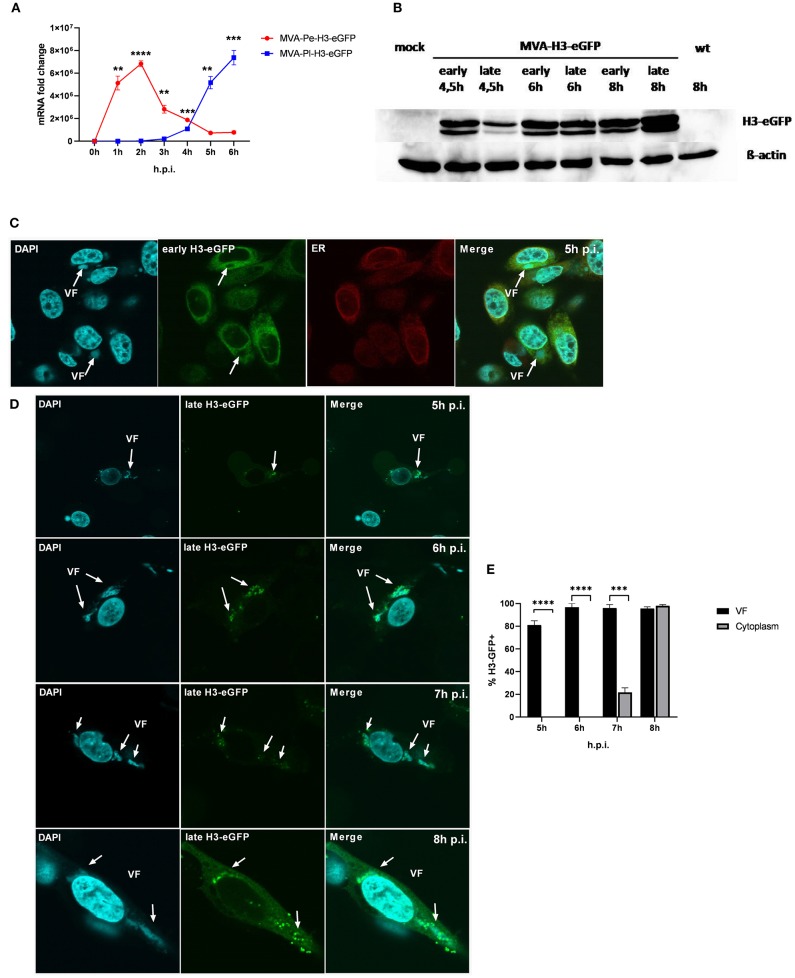
Trapping of late viral proteins (H3-eGFP) in viral factories (VFs). **(A)** mRNA expression kinetics for H3-eGFP in BMDC infected with either MVA-Pe-H3-eGFP (early expression) or MVA-Pl-H3-eGFP (late expression) with MOI 10 determined for 0–6 h p.i. GFP-specific primers were used. Data represent relative expression levels and are displayed as means and SEM (*n* ≥ 3) of results from at least three independent experiments. **(B)** Western blot analysis for protein Synthesis of early and late expressed H3-eGFP. BMDC were infected with MVA-Pe-H3-eGFP (early) or Pl (late) for indicated hours at MOI 10. Mouse anti-GFP Ab was used to stain the H3-eGFP fusion protein. ß-actin was used as loading control. **(C)** Localization of H3-eGFP by CLSM. Early H3-eGFP (green) co-localized with ER at 5 h p.i. in HeLa cells infected with MVA-Pe-H3-eGFP. Nuclei and VFs (blue), ER (red), white arrows indicate VFs. **(D)** H3-eGFP produced late during infection (late H3-eGFP) was detected in VFs at 5 and 6 h p.i. and started to translocate from VFs to cytoplasm at 7 h p.i.. At 8 h p.i. late H3-eGFP was present in VFs and cytoplasm in all infected cells. Nuclei and VFs (blue). eGFP indicates late-H3 location. White arrows point to VFs. CLSM pictures are representative for one of three independent experiments. **(E)** Quantification and statistical analysis for the colocalization of late H3-GFP (% H3-GFP+) with VF (black) and/or cytoplasm (gray) in infected cells. Data are means and SEM (*n* ≥ 30) from three independent experiments, *P < 0.05; **P < 0.01; ***P < 0.001; ****P < 0.0001 (two-tailed Student's *t*-test).

It has been shown that shortly after infection, viral early genes are transcribed in the core of the virion and mRNA is translated in the cytoplasm. In contrast, late viral transcripts are produced and translated in viral factories (VFs) after DNA replication (18). VFs are viral replication sites surrounded by a membrane that is most likely derived from the ER and impose as organelles (35). In order to study a possible impact of the distinct localization of identical viral antigen expressed under control of either early (Pe) or late promoters (Pl) on antigen processing and MHC class I presentation, we used the viral antigen H3 fused to eGFP (H3-eGFP) as a reporter. Since the late antigen H3 is an essential viral membrane protein, we engineered these recMVA to encode an additional copy as a tractable chimerical H3-eGFP fusion protein under the control of either an early (PK1L) or late (P11) promoter (MVA-Pe- or -Pl-H3L-eGFP), respectively (see also Table 1). Viral gene expression (mRNA) and protein synthesis was shown for both recombinant viruses by qPCR (Figure 5A) and western blot analysis for H3-eGFP (Figure 5B). Importantly, late H3-eGFP transcripts were clearly detectable as early as 4 h p.i. and reached comparable amounts to early H3-eGFP (Figure 5A). Likewise, comparable amounts of protein were detected at 6 h p.i. (Figure 5B). Kinetic analysis of the intracellular localization of H3-eGFP by CLSM demonstrated that the localization of the antigen within infected cells was strictly dependent on the time of its expression (Figures 5C–E). At 5 h p.i., early expressed H3-eGFP localized to ER, outside of VFs (Figure 5C). In contrast, when H3-eGFP was expressed late, it was exclusively found in VFs from 5 to 6 h p.i. and started to be additionally visible outside the VFs at 7 h p.i. in about 20% of infected cells (Figures 5D,E). At 8 h p.i, H3-eGFP was still found to be strongly localized in the VFs (Figure 5D, arrows) but also clearly visible outside the VFs in almost all cells (Figures 5D,E). These results are in line with other publications that found vaccinia virus late gene expression and protein synthesis to be restricted to the VFs (18). However, the data also imply that late-expressed H3-eGFP was sequestered in the VFs and could hardly translocate from this compartment within 7 h p.i. This sequestration of late antigen might explain why H3-specific CTL were not activated by infected APC before 8 h post infection (Figure 1B) despite the clear presence of H3-specific transcripts at 4 h p.i. (Figure 1A) and H3 protein at 4.5 h p.i. (Figure 5B).

**Figure 6:**
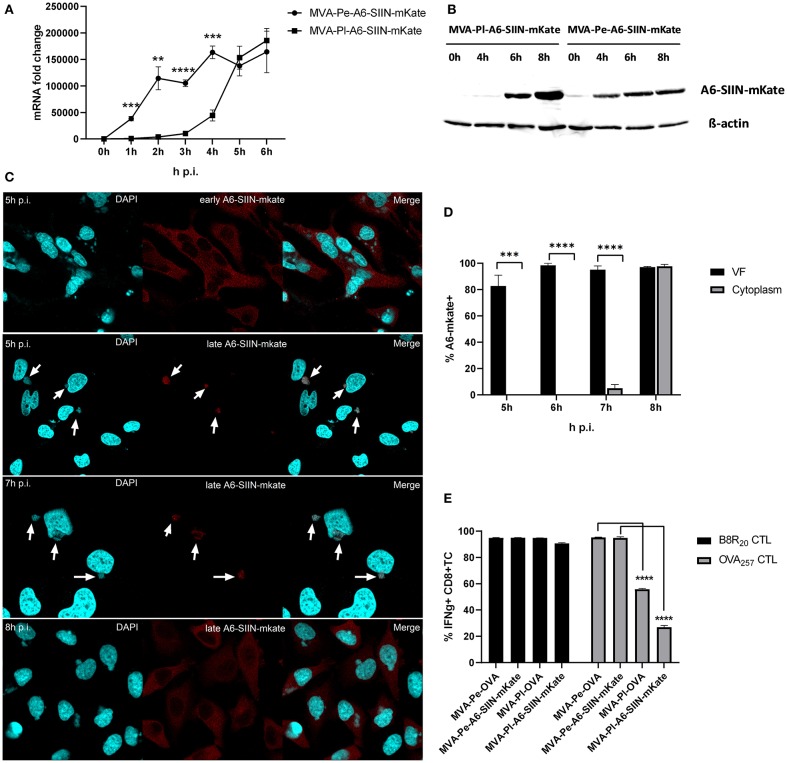
Presentation of viral late antigen A6 to CTL is impaired. **(A)** mRNA expression kinetics for A6-SIIN-mKate in BMDC infected with MVA-Pe-A6-SIIN-mKate (early expression) or MVA-Pl-A6-SIIN-mKate (late expression) with MOI 10 between 0 and 6 h p.i. mKate-specific primers were used. Data represent relative expression levels and are displayed as mean and SEM (*n* ≥ 3) from at least three independent experiments. **(B)** Synthesis of early and late expressed A6-SIIN-mKate determined by western blot analysis. BMDC were infected with MVA-Pe or Pl-A6-SIIN-mKate for indicated hours. Rabbit anti-mKate Ab was used to detect the A6-SIIN-mKate fusion protein. ß-actin was used as loading control. **(C)** Localization of A6-SIIN-mKate by CLSM. Early produced A6-SIIN-mKate (upper panel) was localized in the cytoplasm at 5 h p.i. in HeLa cells infected with MVA-Pe-A6-SIIN-mKate. Late A6-SIIN-eGFP was detectable in VFs at 5 and 6 h p.i and translocated from VFs after 8 h p.i in HeLa cells infected with MVA-Pl-A6-SIIN-mKate. A6-SIIN-mKate (red), Nuclei and VFs (blue).Arrows point to VFs. **(D)** Quantification and statistical analysis for the colocalization of late A6-SIIN-mKate (% A6-SIIN-mKate+) with VF (black) and/or cytoplasm (gray) in infected cells. Data are means and SEM (*n* ≥ 30) from three independent experiments. ^***^P < 0.001; ^****^P < 0.0001 (two-tailed Student's *t*-test). **(E)** Reduced OVA-specific CD8+ T cell activation by late expressed OVA or A6-SIIN-mKate. BMDC were infected with MVA-Pe-OVA (e-ova) or –Pl-OVA (l-ova) or with MVA-Pe-A6-SIIN-mKate (e-A6-SIIN-mKate) or Pl-A6-SIIN-mKate (l-A6-SIIN-mKate) for 8 h. B8-specific T cell line was used as infection control. IFNg production was determined by ICS followed by FACS analysis. Data are means and SEM (*n* ≥ 3) from three independent experiments. ^*^P < 0.05; ^**^P < 0.01; ^***^P < 0.001; ^****^P < 0.0001 (two-tailed Student's *t*-test).

In order to exclude an antigen- or promoter-specific effect, we generated recMVA which expressed another viral antigen under its natural viral late promoter. We choose the viral gene A6L which is expressed late in the MVA viral life cycle (Supplementary Figure 1), although in VACV it is an intermediate gene (11). For monitoring (i) protein synthesis, (ii) intracellular localization and (iii) antigen-specific T cell activation, A6L was fused to the OVA-derived sequence coding for the SIINFEKL oktapeptide (as T cell epitope) followed by the fluorescent protein mKate2 (as marker) under control of either the K1L early promoter (MVA-Pe-A6-SIIN-mKate) or the natural A6L late promoter (MVA-Pl-A6-SIIN-mKate), respectively (see also Table 1). qPCR confirmed the expected early or late A6-SIIN-mKate expression kinetics for both constructs (Figure 6A and Supplementary Figure 1). Notably, the protein amounts of Pl-A6-SIIN-mKate were already higher at 6 h p.i. for the construct expressing the target gene under control of the authentic A6L late promoter as compared to the early promoter construct (Figure 6B). Similarly to H3-eGFP, late expressed A6-SIIN-mKate was fully sequestered in VFs up to 6 h p.i. At 7 h p.i. the late expressed protein started to be visible outside of some VFs (<10%) and only at 8 h p.i. it was clearly detectable outside of all VFs (Figures 6C,D). Importantly, the SIINFEKL (OVA_257_)-specific CTL response was significantly reduced in BMDC infected with MVA-Pl-A6-SIIN-mKate as compared to MVA-Pe-A6-SIIN-mKate at 8 h p.i. (Figure 6E), despite detectable amounts of protein at 4 h p.i. and the abundance of Pl-A6-SIIN-mKate compared to Pe-A6-SIIN-mKate at 5 h p.i. at mRNA level or at 6 h p.i. at protein level. In addition, MVA-Pe-OVA and –Pl-OVA were comparatively used in this setting. Interestingly, MVA-Pl-A6-SIIN-mKate activated OVA_257_-CTL even less efficient than MVA-Pl-OVA (Figure 6E), also supporting poor antigen presentation of a viral late gene product when expressed by its natural promoter. B8-specific T cell activation indicated comparable infection of target cells for all constructs used (Figure 6E). This data supports the idea that the containment of viral proteins in VFs which are otherwise potent antigens for CTL activation when available outside the VFs, interferes with antigen processing for MHC class I presentation.

### Impaired Proteasome Activity Inside VF

After infection, vaccinia virus cores are opened by proteasomal degradation of associated viral core proteins, which have been ubiquitylated before infection presumably within the VF of the original host cell (36). We followed the hypothesis that the presence of active proteasomes within VF would be particularly detrimental for the ubiquitylated core proteins or cores, since this would lead to premature degradation of these structures. Therefore, we anticipated that either proteasomes or at least proteasomal activity might be absent in VF in order not to interfere with viral morphogenesis until ubiquitylated cores will be finally protected by the viral membranous envelope.

**Figure 7:**
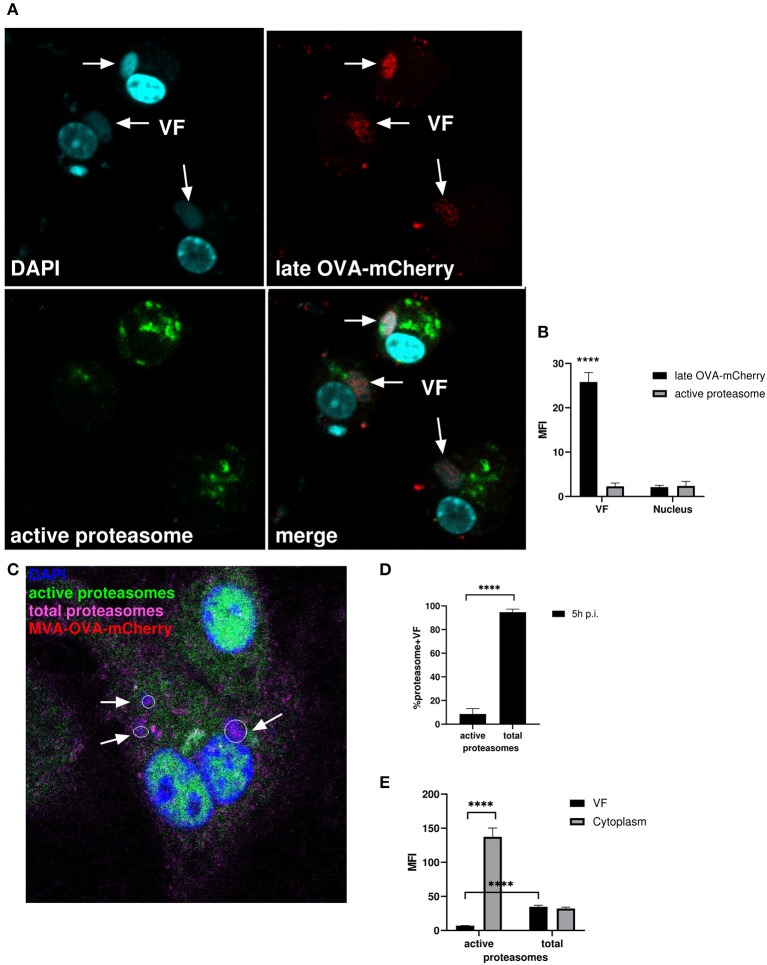
Active proteasomes were absent in VFs. **(A)** CLSM. BMDC were infected for 5 h with MVA-Pl-OVA-mCherry at MOI 10 to mark infected cells and VFs. Active proteasomes (green) were detected in infected cells (red) using Proteasome Activity Probe. Nuclei and VFs were stained by DAPI (blue). White arrows indicate VFs. **(B)** Quantification of **(A)**. Late OVA-mCherry (black bar) or active proteasomes (gray bar) inside VF or nuclei. VF and nuclei area were masked by Fuji auto threshold methods MaxEntropy. MFI is calculated by redirecting those areas to red (OVA-mCherry) or green fluorescence (active proteasome). **(C)** CLSM, higher magnification area from Supplementary Figure 4B. BMDC were infected with MVA-OVA-mCherry at MOI 10 for 5 h. Active proteasomes (green) were detected by using Proteasome Activity Probe. Total proteasomes (pink) were determined with anti-proteasome 20S alpha 1+2+3+5+6+7 antibody. Nuclei and VFs were stained by DAPI (blue). Inactive proteasomes within VF (purple). Arrows mark VFs. **(D,E)** Quantification of (**C** and Supplementary Figure 4B). **(D)** Frequency of VFs positive for total or active proteasomes in MVA-infected BMDC. **(E)** MFI of active proteasomes (green) or total proteasomes (purple) in VF or cytoplasm. MFI is calculated as described in **(B)**. All pictures are representative for one of three independent experiments. **(B,D,E)** Show data as means and SEM (*n* ≥ 30 cells) from three independent experiments, ****P < 0.0001 (two-tailed Student's *t*-test).

We infected BMDC with MVA-Pl-OVA-mCherry expressing OVA fused to mCherry under control of the P11 late promoter (Table 1) to mark infected cells and determined the presence and localization of active proteasomes by a specific probe which needs to be proteasomally processed to be activated and to emit green fluorescence. Interestingly, while late OVA-mCherry appeared in VF after infection as expected, active proteasomes were not detectable within VFs, but accumulated around this compartment in the cytoplasm (Figures 7A,B). HeLa cells infected with MVA-Pe/l-Np-SIIN-eGFP showed similar results (Supplementary Figure 4A). In addition, the lack of proteasomal activity in VFs was not due to the general absence of proteasomes in VFs. In contrast, proteasomes were present in VFs, but seemed to be functionally inhibited (Figures 7C–E and Supplementary Figures 4B,C). The absence of active proteasomes in VFs supports the view that viral proteins expressed in VF may be protected from proteasomal degradation as long as they are retained in this compartment.

In order to corroborate these data, we used a proteasome-sensitive fluorescent reporter (ZsProSensor-1, Clontech), which is a fusion protein of a bright green fluorescent protein with a degradation domain which targets the protein for rapid degradation by the proteasome (Supplementary Figure 5A, upper panel).

HeLa cells were transiently transfected with Proteasome Sensor Vector (pZsProSensor-1) plasmid encoding the gene for Zoanthus sp. Reef coral Green Fluorescent Protein (ZsGreen) fused to the mouse ornithine decarboxylase (MODC) degradation domain (amino acids 410–461). The MODC degradation domain targets the constitutively expressed fluorescent protein for rapid proteasomal degradation. The protein does not accumulate in cells until proteasomes are inhibited which can be detected by an increase in green fluorescence. The cells emitted green fluorescence when proteasomal activity was inhibited leading to sensor accumulation, e.g., after treatment with MG132 (Supplementary Figure 5A, lower panel). After infection, the sensor protein selectively accumulated in VFs (Supplementary Figure 5B) indicating that inactive proteasomes marked by the green probe were present in VF, but not in other compartments e.g., in nuclei [Supplementary Figure 5B, lower picture (profile)]. This finding was corroborated by comparative analysis of mean fluorescent intensities (MFI) between nuclei and VF areas (Supplementary Figure 5C). Here, the MFI for mCherry located in the nuclei of infected cells served as a reference. We detected low to minimal green fluorescence signals in the nuclei and cytoplasm indicating normal proteasomal activity leading to degradation of the sensor. In contrast, we saw high green fluorescent intensity in the VF indicating proteasome inhibition and sensor accumulation. Thus, proteasomes are present in VFs, but in contrast to the surrounding intracellular space, they are functionally inactive leading to accumulation of potential proteasomal substrates.

### Relocating Late Antigen From the VF Can Rescue Antigen Presentation

**Figure 8:**
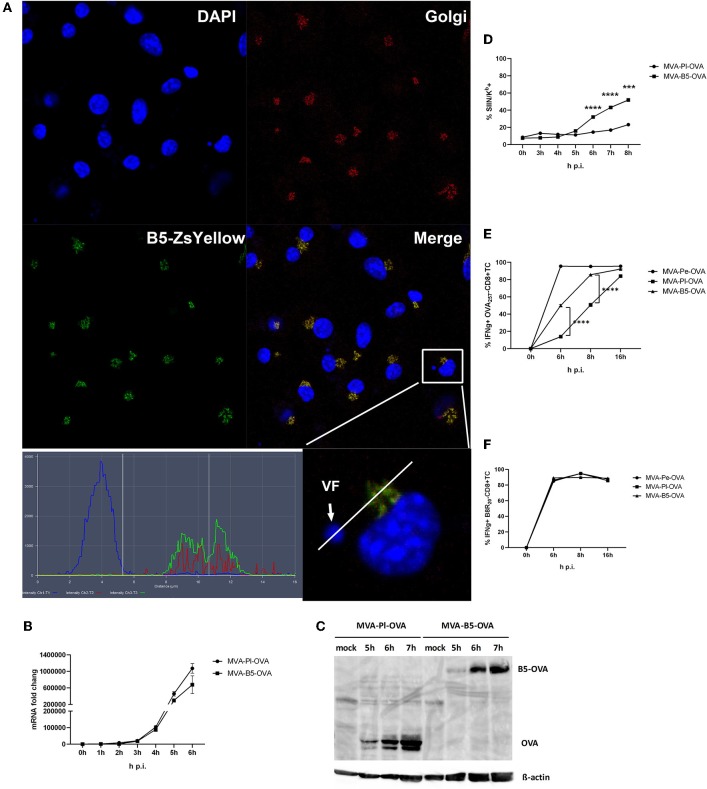
Enhanced antigen processing and presentation as well as CD8+ T cell activation when late antigen is not sequestered in VFs. **(A)** For CLSM, BMDC were infected with MVA-B5-ZsYellow at MOI 10 for 5 h. DAPI (blue), B5-ZsYellow (green) and Golgi (red) stained by GM130 antibody. Picture is representative for one of three independent experiments. Right panel shows the color profile of an infected cell at higher magnification. ImageJ colocalization analysis showed B5 and Golgi have Pearson's Coefficient *R* = 0.548. **(B)** The mRNA expression kinetics for OVA in BMDC infected with either MVA-Pl-OVA (late expression of OVA under control of the P11 promoter) or MVA-B5-OVA (late expression of OVA fused to B5 (B5-OVA) under control of the natural B5 promoter) for 0–6 h p.i. OVA-specific primers were used. Data represent relative expression levels and are displayed as means and SEM (*n* ≥ 3) from at least three independent experiments. **(C)** BMDC were infected with MVA-Pl-OVA or MVA-B5-OVA at MOI 10 for indicated hours. For western blot analysis cell lysates were immunoblotted and OVA detected by using rabbit anti-ova antibody. **(D)** Kinetic analysis of SIINFEKL/K^b^ complex expression at the surface of MVA-B5-OVA or MVA-Pl-OVA infected BMDC (MOI 10). Mouse anti-SIINFEKL/K^b^ APC antibody was used. Frequency of SIINFEKL/K^b^+ cells (% SIIN-K^b^+ cells) is shown. **(E,F)** BMDC were infected with MVA-Pe-OVA, MVA-Pl-OVA or MVA-B5-OVA at MOI 10 for indicated hours and afterwards co-incubated with OVA- or B8-specific CD8+ T cells. IFNg production (ICS followed by FACS analysis) is shown. Data are means and SEM (*n* ≥ 3) from three independent experiments. ***P < 0.001, ****P < 0.0001 (two-tailed Student's *t*-test).

B5 is a viral late gene for MVA (Supplementary Figure 1), but has been reported to be expressed early and late for VACV (22). It is part of the envelope of the virion and after infection primarily located in the Golgi (37). We confirmed its colocalization to the Golgi after MVA infection of BMDC using a recMVA expressing B5 fused to Zs Yellow under control of the natural B5 promoter (Table 1) at 5 h p.i. (Figure 8A). The shuttling to the Golgi in the VF after expression appears to be very stringent and rapid as we failed to detect the fusion protein in the VF at 5 h p.i. (Figure 8A) as well as at earlier and later time p.i. (data not shown).

Next we were interested, if we might improve the poor CD8+ T cell activation against late antigens by minimizing the retention time in VFs and by changing the intracellular location. We took advantage of a pair of MVA constructs, both expressing the model antigen OVA under control of a late promoter, but one antigen variant would escape the VF as early as it is synthesized [B5 fused to OVA expressed under control of the natural late B5 promoter (B5-OVA)] while the other stays in the VF for several hours as we have shown earlier [OVA expressed under control of the late promoter (Pl-OVA)]. Infection of BMDC using MVA-B5-OVA resulted in a comparable OVA-specific mRNA expression profile as with MVA-Pl-OVA as determined by qPCR (Figure 8B). In addition, both vectors produced comparable amounts of OVA proteins with similar kinetics (Figure 8C). Despite the above similarities, the appearance and frequency of SIINFEKL/K^b^ complexes expressing cells (Figure 8D), the density of SIINFEKL/K^b^ complexes at the cell surface (Supplementary Figure 3B) and the OVA-specific CD8+ T cell activation (Figure 8E) were significantly enhanced after infection of BMDC with MVA-B5-OVA as compared to MVA-Pl-OVA. At 8 h p.i. MVA-B5-OVA allowed for almost comparable T cell activation levels as MVA-Pe-OVA (expressing OVA early) which was used as a positive control (Figure 8E). Notably, B8R_20_-specific T cell activation was comparable for all constructs used in this experiment (Figure 8F) demonstrating comparable infection rates of target cells.

Taken together, VFs seem to efficiently retain MVA-derived viral and recombinant antigens when they were expressed late during infection. All late proteins were initially produced in VFs and as demonstrated, some proteins investigated here were found in this compartment for several hours. In contrast, identical proteins were localized in the cytoplasm or ER when expressed early. Since active proteasomes were absent in VFs, we conclude that the initial expression and continuing sequestration of late antigens in VFs protected these proteins from proteasomal degradation. This accounts for the delayed or even absent antigen processing and MHC class I presentation of these antigens and resulted in poor protection in our challenge model due to impaired memory CTL activation.

## Discussion

MVA is a broadly used vaccine vector (6, 38). In order to improve vaccine immunogenicity in the past, particularly with regard to CD8+ T cell responses, efforts were undertaken to enhance the promotor efficacy for transgene expression. Most researchers focused on the improvement of early (16, 39, 40) or early/late promoters (14) since first evidence came up that late promoters are not as effective to induce CD8+ T cell responses despite strong transgene expression (41). Since then, late antigens were regarded as a good source for antibody responses but poor in activating T cells.

The aim of this study was to elucidate the phenomenon that MHC class I-restricted presentation of epitopes derived from MVA late gene products as well as reactivation of cognate CTL *in vitro* or memory CTL *in vivo* is substantially delayed or even completely absent (26). Understanding the mechanism behind this deficiency may help to improve MVA-mediated adaptive immunity by simultaneously targeting efficient T cell and antibody responses.

We found that in contrast to early viral epitopes, the impaired presentation profile of late viral epitopes to CTL did not correlate with MVA late gene expression kinetics. Late antigens were abundant, but not processed and presented. We performed a CLSM-based analysis of the localization of MVA gene products in specific subcellular compartments during the course of infection, which allowed us to provide a mechanistic explanation for the impaired presentation of late viral antigens to CTL. We show that the initial localization of late antigens in so-called viral factories (VFs) is further maintained for up to 3 h for some of the antigens tested. The containment in VFs dramatically altered the fate of late antigens in infected target cells for viral T cell epitope processing and presentation. We demonstrate that proteasomes were present in VFs, but in a proteolytically inactive state which prevents degradation of antigens in this compartment and most likely efficiently interferes with antigen processing for MHC class I.

Various factors contribute to the epitope specificity and immunodominance pattern of virus-induced T cell responses during infection, such as MHC binding affinity, efficiency of cellular antigen processing to generate the relevant peptides as well as the TCR repertoire and T cell avidity (42). Some viruses inhibit antigen presentation by targeting the MHC class I machinery (43). We could show that the antigen processing and presentation machinery in MVA-infected cells was fully functional and efficient even at late time points in the course of infection. However, for late antigen-derived epitopes, the appearance of peptide-loaded MHC class I complexes at the cell surface as well as the activation of specific T cell lines took place with considerable delay. Therefore, we hypothesized that the distinct localization of early or late antigens might have an impact on their processing.

MVA is a highly attenuated VACV strain with a known safety profile for clinical usage as a vaccine vector (4, 5). VACV is characterized by its replication in the cytoplasm where it forms special organelles called VFs, which are usually located close to the nucleus. As factories form besides the nucleus, newly synthesized ER membranes are recruited to the replication sites and will eventually form an almost completely sealed envelope around the VF (35). Some viral membrane proteins synthesized within VFs are selected to be fused with viral crescent membranes (such as H3) or to translocate to the Golgi (such as B5) (44). It is however still not known, how viral antigens or viral particles are retained in or released from VFs (45), although it may be influenced by some translation factors needed in VFs (46) or by functional interaction with other viral proteins (47).

Up to now, a possible impact of VFs on antigen processing and presentation for MVA derived recombinant antigens has not been investigated. One study on VACV demonstrated that the recombinant antigen β-gal was also localized in VFs at 5 h p.i. in non-professional APC and was not cross-presented *in vivo* when expressed late (24). Interestingly, direct presentation of late antigens by BMDC was excluded by the study based on lack of β-gal activity in these cells.

In the current study, early produced antigens were localized in the cytosol or at intracellular membranes depending on their respective targeting signals, which allowed efficient antigen presentation. H3 owns a membrane targeting signal which targeted H3-eGFP to the ER when it was produced early. Yet, H3-eGFP was present in VFs for an extended time period as a late-expressed antigen which was accompanied by delayed antigen presentation to CTL. Interestingly, the retention time of late antigens in VFs varied considerably and was influenced e.g., by targeting signals to structures outside the factory as shown for B5 which could be detected in the Golgi early on. The delivery of OVA fused to B5 (B5-OVA) as late antigen enhanced OVA-specific CD8+ T cell activation significantly. Therefore, the usage of targeting signals in recombinant proteins produced late by MVA which alter the antigen localization early after expression may help to circumvent viral evasion from CTL recognition and further enhance immunogenicity.

Furthermore, the long retention time of these antigens in VFs was associated with low processing by the proteasomal antigen processing machinery, because active proteasomes were absent in VFs and congruously, antigens in VFs not degraded. Proteasomal degradation represents the major source of peptide ligands for MHC class I presentation. Proteasomal function has been implicated in the regulation of viral trafficking, replication, egress, and immune evasion (48). Currently we have only a working model for the proteasomal impairment in VFs in our study. Most likely the virus encodes a yet unidentified gene product that is able to either directly or indirectly interfere with proteasomal activity in the VF. This hypothesis is based on the fact that infectious virions carry a pre-ubiquitinated core which is essential to release the viral genome from the core by proteasomal degradation in the cytoplasm of the newly infected cell after entry (36). This step is a prerequisite for replication of the viral DNA in VACV and also for intermediate and late gene expression in MVA infected cells (49). Since ubiquitination of the cores takes place in VFs, the presence of active proteasomes would be detrimental in this organelle and most likely lead to premature proteasomal destruction of ubiqutinated core proteins. Thus, the virus may truly benefit from or even require inactive proteasomes in VFs for productive infection. Other researchers have observed the accumulation of ubiquitins in colocalization with other proteins within poxvirus replication sites (50). In this respect, one may anticipate that late viral proteins already marked by ubiquitin moieties within the VFs must be protected from proteasomal degradation until the replication cycle has been completed and intact virus particles have been formed. In addition, some antigens are more prone to proteasomal degradation than others which involve e.g., stability, function or location within the cell (48, 50, 51). These differences may contribute to the variability in efficacy as well as kinetics of antigen presentation for individual late antigens that we observed.

The primary response after MVA vaccination requires direct priming by infected APC as well as cross-priming via non-infected APC for optimal CD8+ T cell responses and allows for the induction of both, early and late antigen-specific CTL (25, 52). Recall responses namely the boosting of pre-existing memory T cells with MVA may depend more on direct presentation by infected APC since the T cell pool is critically shaped by cross-competition of CTL at the level of infected APC (26, 53). This results in a dramatic change in the immunodominance hierarchy favoring the expansion of CTL specific for early antigens. However, more experimental data are required to substantiate this idea. We demonstrate here that after a secondary MVA vaccination the reactivation and expansion of memory CD8+ T cells specific for OVA were almost absent, when the antigen was expressed late. Importantly, this failure in boosting the primary response resulted in complete loss of protection in an Lm-OVA challenge model despite pre-existing ova-specific memory CTL. With respect to human vaccine studies, the ability of human DCs to allow for late gene expression seems to be strongly dependent on the DC subset or more accurately for experimental use dependent on the progenitor cell and the method by which DC have been generated (54). In two studies, MVA- or WR-infected human blood monocyte-derived DC generated from adherent monocytes treated with GMCSF and IL4 for 7 days and further matured to CD83+ mDC with TNFa IL-1ß IL-6 PGE2 failed to allow for late gene expression (8, 55). In contrast, potent late gene expression was reported in human DC infected with MVA expressing *LacZ* under control of the viral P11 late promoter. In this study, CD34+ progenitor DC were mobilized from the bone-marrow in patients, sorted and further matured to DC *in vitro* using GMCSF, TNFa, SCF and flk2/flt-3 for 10–14 days (56). Since DC gene expression profiles are dependent on their environment (57), different DC subtypes in humans originating from blood, tissue or bone marrow will most likely differ in their capability and efficacy to express vaccinia virus late genes. Future studies in humans will be needed to identify submissive DC subsets or preparations.

In summary, the timing of viral antigen production and the subsequent subcellular localization had a strong impact on the processing and presentation of MVA-delivered antigens on MHC class I and subsequent CTL activation. Particularly, the prolonged sequestration of late antigens in VFs in which proteasomal activity was significantly reduced, accounted for the delayed or even absent presentation to CTL. As a consequence, for the immunogenicity of MVA as a vaccine vector *in vivo*, it may be anticipated that the immunodominance pattern found after boost vaccinations with MVA marked by reduced proliferation of T cells specific for late viral proteins is not only shaped by cross-competition of T cells specific for early antigens. It seems also to be directly influenced by the transient deprivation of late antigens for further processing and presentation on MHC class I due to their isolation in VFs. Since recMVA are preferably used as boost vectors in heterologous prime/boost regimens (4, 58), this is a relevant issue and gives important information for current as well as optimized vaccine design.

## Data Availability Statement

All datasets generated for this study are included in the article/Supplementary Material.

## Ethics Statement

The animal study was reviewed and approved by District Goverment of Upper Bavaria, Germany and North Rhine-Westphalia State Environment Agency-LANUV, Germany.

## Author Contributions

ST and RT conducted experiments and analyzed the data. RT managed cell lines and generated all the recMVA. DB provided tetramers and contributed to experiments. MW and HS contributed to qRT-PCR experiments. ST and ID designed the study, interpreted the data, and wrote the manuscript.

### Conflict of Interest

The authors declare that the research was conducted in the absence of any commercial or financial relationships that could be construed as a potential conflict of interest.
