# Supplementary material for: Sequestration of Late Antigens Within Viral Factories Impairs MVA Vector-Induced Protective Memory CTL Responses
Source: Front Immunol. 2019 Dec 4;10:2850. doi: 10.3389/fimmu.2019.02850 (PMC6904312; doi:10.3389/fimmu.2019.02850)
Supplement: Supplementary file 1 [file Data_Sheet_1.PDF]

Supplementary Material

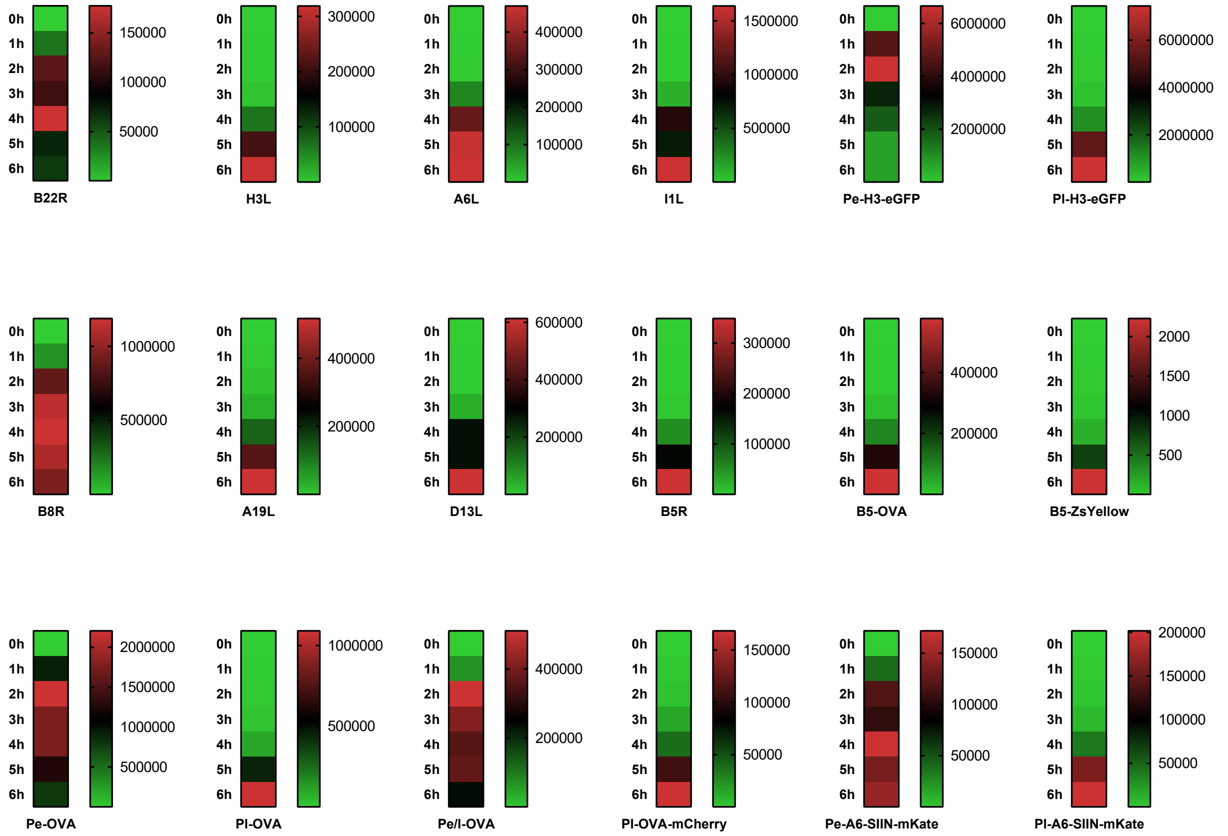

Supplementary Figure 1. Cluster analysis of MVA gene expression by qRT-PCR. The mRNA expression kinetics for MVA genes in infected BMDC (MOI 10) were determined between 0–6h post infection (p.i.). Single gene-specific heat maps are shown. The red and green colors indicate high and low expression, respectively. Data are represented as relative expression levels and are displayed as mean (n>=3). The qRT-PCR was performed in duplicates and repeated in at least three independent experiments.

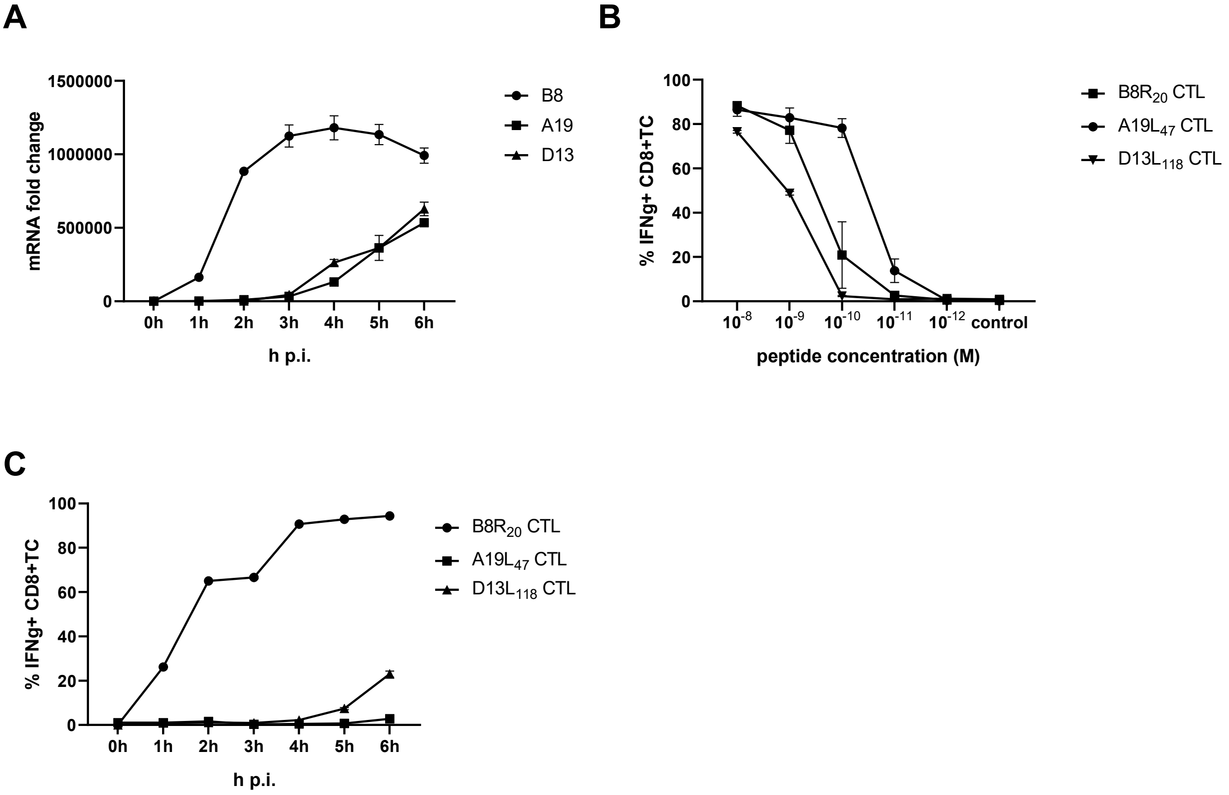

Supplementary Figure 2. Antigen presentation of late viral proteins is significantly delayed and reduced. **(A)** qRT-PCR. Gene expression profile for B8, A19, D13 in BMDC infected with MVA-wt (MOI 10) from 0h to 6h post infection (h p.i.). **(B)** Peptide titration showed similar avidity for all CTL. BMDC were loaded with indicated peptides at concentrations ranging from 10-8 M to 10-12 M or with irrelevant MHC class I binding peptides at 10-8 M as negative control. ICS for IFNg production. **(C)** BMDC were infected with MVA-wt at MOI 10 for indicated hours to stimulate IFNg production in MVA-specific T cells (B8R$_{20}$, A19L$_{47}$, D13L$_{118}$). In contrast to early produced antigens (B8), recognition of late antigens (A19, D13) was massively reduced in infected cells. All data are means and SEM (n>=3) from three independent experiments.

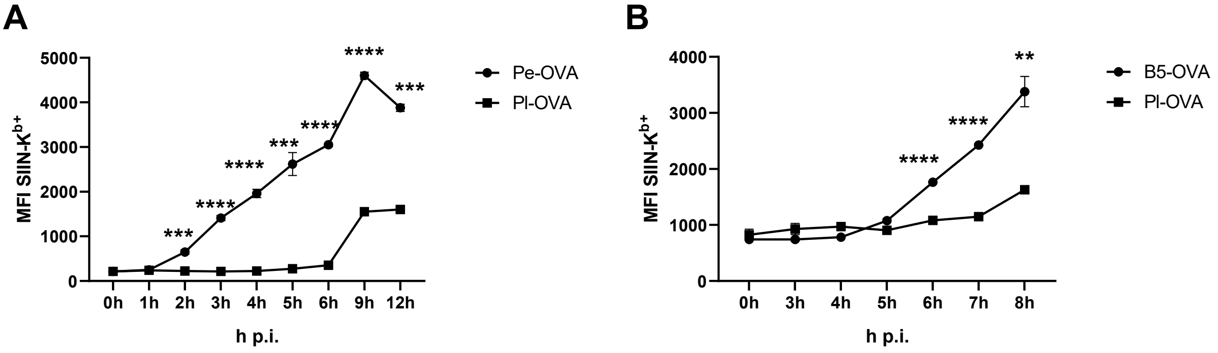

Supplementary Figure 3. Presentation of SIINFEKL/K^{b}. **(A)** Kinetic analysis of SIINFEKL/K^{b} complex expression at the surface of MVA-Pe-OVA or MVA-Pl-OVA infected BMDC. Mouse anti-SIINFEKL/K^{b} APC antibody was used. **(B)** Kinetic analysis of SIINFEKL/K^{b} complex expression at the surface of MVA-B5-OVA or MVA-Pl-OVA infected BMDC. Mouse anti-SIINFEKL/K^{b} APC antibody was used. MFI of SIINFEKL/K^{b}+ cells is shown. All data are means and SEM (n>=3) from three independent experiments. * P< 0.05; ** P< 0.01; *** P< 0.001 **** P< 0.0001 (two-tailed Student's t test).

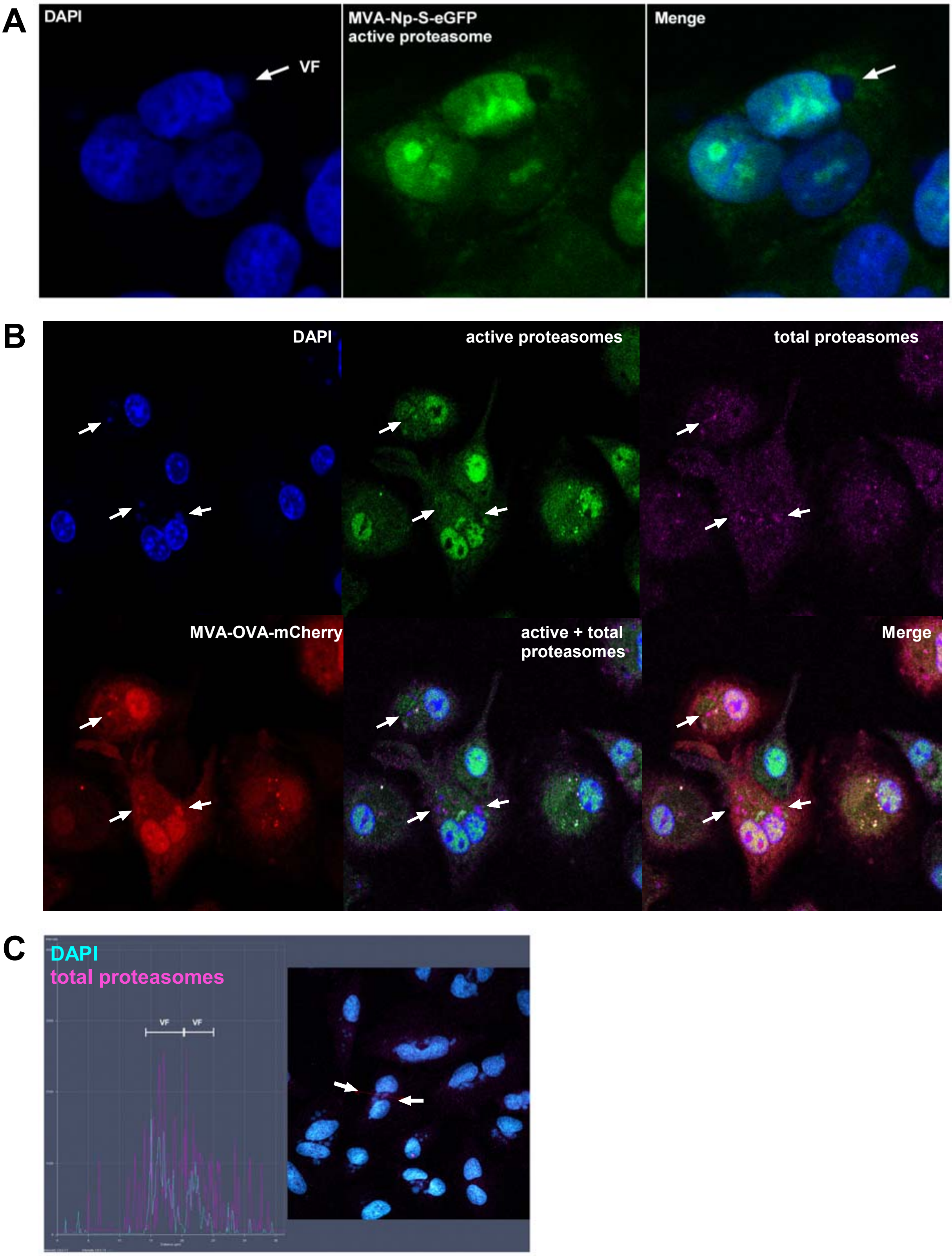
A
DAPI
VF
MVA-Np-S-eGFP
active proteasome
Menge
B
DAPI
active proteasomes
total proteasomes
MVA-OVA-mCherry
active + total
proteasomes
Merge
C
DAPI
total proteasomes
VF
VF

Supplementary Figure 4. Active proteasomes were absent in VFs. **(A)** HeLa cells were infected with MVA-Pe/l-Np-SIIN-eGFP (Table 1) at MOI 10 for 5h to mark infected cells. Infected cells display intensive green nuclei due to the nuclear targeting signal of Np. Active proteasomes appear green in the cytoplasm. Nuclei and VFs were stained by DAPI (blue). White arrow indicates VF. **(B)** Split channels (corresponds to zoomed area shown in Figure 7C). BMDC were infected with MVA-Pe/l-OVA-mCherry (Table 1) at MOI 10 for 5h to mark infected cells (Red). Active proteasomes (green) were detected using Proteasome Activity Probe. Total proteasomes (pink) were determined with anti-proteasome 20S alpha 1+2+3+5+6+7 antibody. Nuclei and VFs were stained by DAPI (blue). White arrows indicate VFs. **(C)** HeLa cells were infected with MVA-wt at MOI 10 for 5h. After fixation, cells were stained for total proteasome (pink). Nuclei and VFs were stained by DAPI (blue). The profile (left) shows the intensity of total proteasomes (pink) in the two VF areas (blue) by a red cutting line marked by two arrows. All pictures shown are representative one of the three independent experiments.

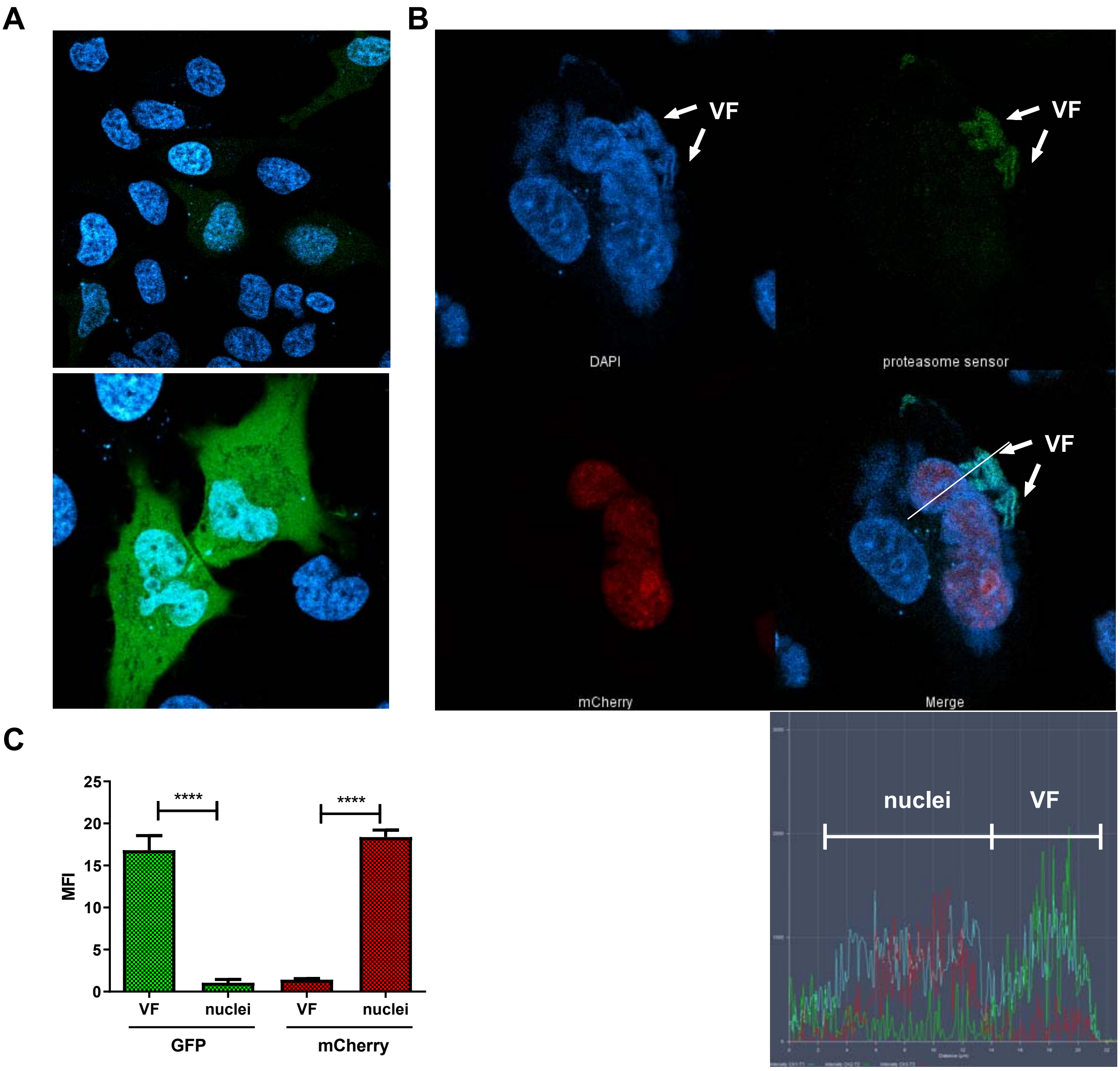

Supplementary Figure 5. Selective inhibition of proteasomal activity in VFs. **(A)** HeLa cells were transfected with ZsProSensor-1 and treated (lower panel) or not (upper panel) with MG132 for 5h. ZsProSensor-1 is a fusion protein of GFP with a degradation domain which targets the protein for rapid degradation by the proteasome. **(B-C)** HeLa cells transfected with proteasome sensor ZsProSensor-1 were infected with VACV-Pe/l-Np-SIIN-mCherry (Table 1) at MOI 10 for 5h to mark infected cells. Infected cells display red nuclei due to the nuclear targeting signal of Np. Blue (DAPI) for nuclei and VF; green (GFP) due to accumulation of the sensor (no proteasomal degradation). White arrows mark VFs. Lower picture (profile) shows the distinct fluorescence intensities in nuclei and VF areas along the white cutting line (upper picture). All pictures are representative for one of three independent experiments. **(C)** Quantification of (B). MFI of GFP or mCherry calculated for VF or nuclei areas. Data are means and SEM from 3 independent experiments. **** $P<0.0001$ (two-tailed Student’s t test).
